# Neutrophil-to-Lymphocyte Ratio, Platelet-to-Lymphocyte Ratio, and Glucose Transporter 1 (GLUT-1) as Predictors of Insulin Resistance in Patients With Rheumatoid Arthritis: A Cross-Sectional Study

**DOI:** 10.7759/cureus.106772

**Published:** 2026-04-10

**Authors:** Diptimayee Upadhyay, Subhashree Ray, Pragya Panda, Rajesh Kumar Bhola, Aswini Kumar Swain, Minushree Pasayat, Florina Swain

**Affiliations:** 1 Department of Biochemistry, Institute of Medical Sciences and SUM Hospital, Siksha ‘O’ Anusandhan (Deemed to be University), Bhubaneswar, IND; 2 Department of Pathology, Institute of Medical Sciences and SUM Hospital, Siksha ‘O’ Anusandhan (Deemed to be University), Bhubaneswar, IND

**Keywords:** glucose transporter 1, insulin resistance, neutrophil-to-lymphocyte ratio, platelet-to-lymphocyte ratio, rheumatoid arthritis

## Abstract

Background and aim: Rheumatoid arthritis is a chronic systemic inflammatory disorder associated with increased cardiometabolic risk, including insulin resistance. Persistent immune activation and metabolic reprogramming of inflammatory cells are considered key mechanisms linking inflammation to impaired insulin signaling. Hematological inflammatory indices and glucose transporter 1 (GLUT-1) expression may serve as accessible biomarkers of this interaction. This study aimed to evaluate the neutrophil-to-lymphocyte ratio (NLR), platelet-to-lymphocyte ratio (PLR), and GLUT-1 expression on circulating immune cells as predictors of insulin resistance in patients with rheumatoid arthritis. It also assessed their association with inflammatory status, compared these biomarkers between patients with and without metabolic involvement, and determined their correlation with insulin resistance indices to identify independent predictors of metabolic dysfunction.

Methods: A cross-sectional study was conducted among 100 patients with rheumatoid arthritis. Clinical evaluation and fasting laboratory investigations were performed. Insulin resistance was estimated using the homeostatic model assessment of insulin resistance (HOMA-IR). NLR and PLR were calculated from complete blood counts. Flow cytometry was used to quantify GLUT-1 expression on monocytes and granulocytes. Statistical analyses included correlation and multivariate regression models.

Results: Patients with insulin resistance demonstrated significantly higher NLR, PLR, and GLUT-1 expression compared to those without insulin resistance. HOMA-IR showed positive correlations with these biomarkers. Multivariate analysis identified NLR and monocyte GLUT-1 expression as independent predictors of insulin resistance.

Conclusion: Elevated inflammatory indices and increased GLUT-1 expression in immune cells are significantly associated with insulin resistance in rheumatoid arthritis, supporting their potential role in metabolic risk stratification.

## Introduction

Rheumatoid arthritis (RA) is a long-lasting autoimmune inflammatory disease whereby there is a continuous synovial inflammation, progressive joint erosion, and systemic flare-ups that do not lie within the musculoskeletal system [1]. Systemic inflammatory environment of RA is complicative of several extra-articular disorders such as cardiovascular disease, metabolic syndrome, and insulin resistance [2]. The body of evidence showing that metabolic disturbances are not incidental to RA but are closely linked to chronic immune activation and cytokine dysregulation is growing [3]. Insulin resistance (IR) is a disease condition in which peripheral tissues show decreased sensitivity to insulin, leading to an inability of cells to take up glucose and to insulin resistance [4]. It has been identified that chronic low-grade inflammation is a key process in the pathogenesis of insulin resistance in autoimmune diseases [5]. The proinflammatory cytokines disrupt the insulin receptor signaling pathways and stimulate oxidative stress, thus implicating them in metabolic dysfunction [6]. Persistent inflammation in RA can enhance these processes and speed up metabolic abnormalities [7].

RA patients have a higher rate of insulin resistance than the general population, even in the absence of conventional metabolic risk factors [8]. This association implies that inflammatory pathways associated with disease have a direct effect on metabolic impairment [9]. RA has been implicated with insulin resistance that has led to cardiovascular morbidity and worse long-term outcomes [10]. Timely diagnosis of the metabolic abnormalities is therefore very important in the overall management of the disease [11]. There has been increased interest in inflammatory biomarkers derived from standard hematological measures as convenient indicators of systemic inflammatory burden [12]. The neutrophil-to-lymphocyte ratio (NLR) reflects the balance between innate and adaptive immune responses and has been suggested to reflect inflammatory activity in most chronic diseases [13]. High levels of NLR have been linked with worsening of the disease and poor prognosis in inflammatory diseases [14]. The increased NLR values have been linked with the active states of the disease and systemic complications in RA [15].

Another available inflammatory index is the platelet-to-lymphocyte ratio (PLR), characterized by platelet activation and lymphocyte suppression under inflammatory stress [16]. Platelets aid in immune regulation and the release of cytokines, and they thus have an effect on inflammation and vascular behavior [17]. Chronic autoimmune disorders have been linked to increased PLR, inflammatory activity, and metabolic abnormalities [18]. The appeal of these indices in clinical practice is that they are cost-effective and can be judged by taking normal laboratory tests [19]. Immune cells have also been found to undergo metabolic reprogramming as a significant aspect of chronic inflammatory diseases [20]. Glucose transporter 1 (GLUT-1) is important in promoting the uptake of glucose in cells, especially immune cells that are activated [21]. The increased expression of GLUT-1 helps to promote the activity of glycolytic metabolism needed to sustain prolonged inflammatory processes [22]. Disturbed GLUT-1 expression has been reported in autoimmune diseases and is considered an indicator of immune cell responses and metabolic changes [18]. Raised levels of GLUT-1 in circulating immune cells are a possibility of a connection between inflammation and impaired glucose metabolism in RA [23]. Increased glucose uptake by inflammatory cells may add to the metabolic imbalance in the system and insulin resistance [24]. The connection between immune activation and metabolic signaling pathways underscores the use of a combination of inflammatory and metabolic indicators when assessing disease [25].

Although there has been increased awareness of the relationship between RA and insulin resistance, there are no standardized biomarkers to identify metabolic dysfunction in such patients at an early stage. Most of the current studies have been conducted on traditional inflammatory markers without the inclusion of hematological ratios and cellular metabolic markers. There is little evidence on the concurrent measurement of NLR, PLR, and GLUT-1 mRNA in comparison to insulin resistance in populations with RA. These biomarkers have not been well characterized in terms of their independent predictive value for metabolic impairment. The lack of integrative biomarker models prevents the stratification of patients who are at increased risk of developing insulin resistance and its associated complications. To improve the risk assessment strategies, they need to be identified with reliable, non-invasive, and reproducible markers. The improved appreciation of the inflammatory-metabolic interface can help intervene earlier and manage the disease in the best possible way.

## Materials and methods

Study design and participants

This is a cross-sectional analytical study conducted to examine inflammatory and metabolic biomarkers associated with insulin resistance in patients with rheumatoid arthritis (RA). One hundred consecutive patients visiting the outpatient rheumatology clinic were recruited and stratified into two equal groups with or without diabetes mellitus. Rheumatoid arthritis was diagnosed according to the American College of Rheumatology/European League Against Rheumatism (ACR/EULAR) classification criteria. Clinical assessment included a detailed medical history, disease duration, and evaluation of disease activity. All participants were informed and gave written consent before joining the study.

Inclusion and exclusion criteria

The eligible participants were older individuals of the specified age, had a known diagnosis of RA, and, at the time of recruitment, had been clinically stable for at least a specified period. In the present study, recent rheumatoid arthritis was defined as a disease duration of less than two years from the time of diagnosis. This definition is consistent with prior clinical studies that categorize early rheumatoid arthritis within a one to two-year window, reflecting the phase of active immunological and inflammatory progression. However, some definitions use shorter durations (e.g., <6 months or <2 weeks); the broader timeframe was adopted to ensure an adequate sample size and to capture clinically diagnosed cases in routine practice settings. This definition is also supported by established literature, in which early rheumatoid arthritis is commonly defined as occurring within the first one to two years after disease onset, reflecting a critical window of immunological activity and therapeutic intervention (e.g., American College of Rheumatology/European League Against Rheumatism recommendations and prior cohort studies). Therefore, the chosen cutoff is both clinically relevant and methodologically appropriate for capturing early disease dynamics [26,27].

Patients with comorbid diabetes were grouped accordingly. Exclusion criteria were aimed at reducing confounding metabolic or inflammatory states and entailed pregnancy, other autoimmune diseases, secondary causes of insulin resistance, and recent corticosteroids. These inclusion criteria provided a fairly homogeneous group of participants in which inflammatory and metabolic biomarkers could be assessed without significant external factors that would affect insulin sensitivity or immune activity.

Biomarker assessment

The venous blood samples were taken following an overnight fasting under standard conditions. Quantitative data of parameters of complete blood count were recorded based on automated hematology analyzers, and the NLR and PLR were calculated based on the counts of differentials. The expression of GLUT-1 was measured by flow cytometry using monoclonal antibodies against the surface transport protein on circulating immune cells, including monocytes and granulocytes. The intensity of the fluorescence was obtained to identify the relative levels of expression. Insulin resistance was estimated using the homeostasis model assessment approach, and fasting glucose and insulin levels were measured using standardized laboratory techniques.

Statistical analysis

Statistical analysis was performed using SPSS Statistics software version 25 (Armonk, NY: IBM Corp.). Continuous variables were tested for normality using the Shapiro-Wilk test. Normally distributed variables were expressed as mean±standard deviation (SD), whereas categorical variables were expressed as frequencies and percentages. Comparisons between rheumatoid arthritis patients with and without diabetes were performed using the independent-samples Student’s t-test for continuous variables and the chi-square (χ²) test for categorical variables. The association between inflammatory biomarkers (neutrophil-to-lymphocyte ratio, platelet-to-lymphocyte ratio, and GLUT-1 expression) and insulin resistance (homeostatic model assessment for insulin resistance {HOMA-IR}) was evaluated using Pearson’s correlation coefficient (r). No additional multivariable adjustments were performed for potential confounding factors such as dietary habits, physical activity, medication use, or lifestyle variables due to the unavailability of complete data. The absence of adjustment for these variables may have introduced residual confounding, potentially leading to an overestimation of the association between inflammatory biomarkers and insulin resistance. For instance, factors such as sedentary lifestyle or corticosteroid use may independently increase insulin resistance, thereby influencing the observed relationships. However, strict exclusion criteria were applied to minimize major confounding effects.

No additional multivariable adjustments were performed for potential confounding factors such as dietary habits, physical activity, medication use, or lifestyle variables due to the unavailability of complete data. However, key exclusion criteria were applied to minimize major confounders. The lack of adjustment for residual confounders may have influenced the observed associations and should be considered while interpreting the results.

Ethical considerations

Before the commencement of the trial, the protocol was approved by the institutional ethics committee. It was carried out in compliance with the established ethical guidelines and the tenets stated in the Declaration of Helsinki. The participants were made aware of the study aims, methods, and risks, which were to be involved in before giving written consent. The research process ensured the confidentiality of the patient data. There was no experimental intervention, and all laboratory tests were done by standard clinical diagnostic procedures so that they could be safe, as well as to be as methodologically transparent as possible.

## Results

Demographic and clinical characteristics

There were 100 patients with rheumatoid arthritis, and an equal number of them were assigned to both of the groups, depending on whether they had diabetes and insulin resistance or not. The age distribution and the proportion of sex predominance did not differ significantly between the two groups, and the baseline demographic features were comparable. There was also similarity in disease activity scores and body mass index among groups. The coexisting diabetes patients showed a considerably extended period of RA, which suggests the potential correlation of chronic inflammatory load and metabolic regulation.

Patients with rheumatoid arthritis and diabetes demonstrated a significantly longer duration of rheumatoid arthritis compared to those without diabetes (6.8±2.4 vs. 4.3±1.9 years, p=0.001), suggesting that prolonged inflammatory burden may contribute to the development of metabolic dysfunction. The mean duration of diabetes was 5.2±2.1 years. Table 1 presents the demographic characteristics of patients with rheumatoid arthritis with and without diabetes. Table 2 shows that the duration of rheumatoid arthritis was significantly higher in patients with diabetes compared to those without, while the duration of diabetes was reported only in the diabetic group.

**Table 1:** Demographic characteristics of rheumatoid arthritis patients with and without diabetes. Continuous variables were compared using the independent-samples Student’s t-test, while categorical variables were analyzed using the chi-square (χ²) test. Data are expressed as mean±SD or n (%). A p<0.05 was considered statistically significant. RA: rheumatoid arthritis

Characteristics	RA with diabetes (n=50)	RA without diabetes (n=50)	Test statistic	p-Value
Age (years)	56.7±10.2	53.9±10.6	t=1.35	0.18
Gender (female)	36 (72%)	34 (68%)	χ²=0.19	0.66

**Table 2:** Disease duration and clinical characteristics of study participants. Data are expressed as mean±SD. A p<0.05 was considered statistically significant. RA: rheumatoid arthritis

Variables	RA with diabetes (n=50)	RA without diabetes (n=50)	p-Value
Duration of RA (years)	6.8±2.4	4.3±1.9	0.001
Duration of diabetes (years)	5.2±2.1	-	-

Neutrophil-to-lymphocyte ratio (NLR)

**Figure 1:**
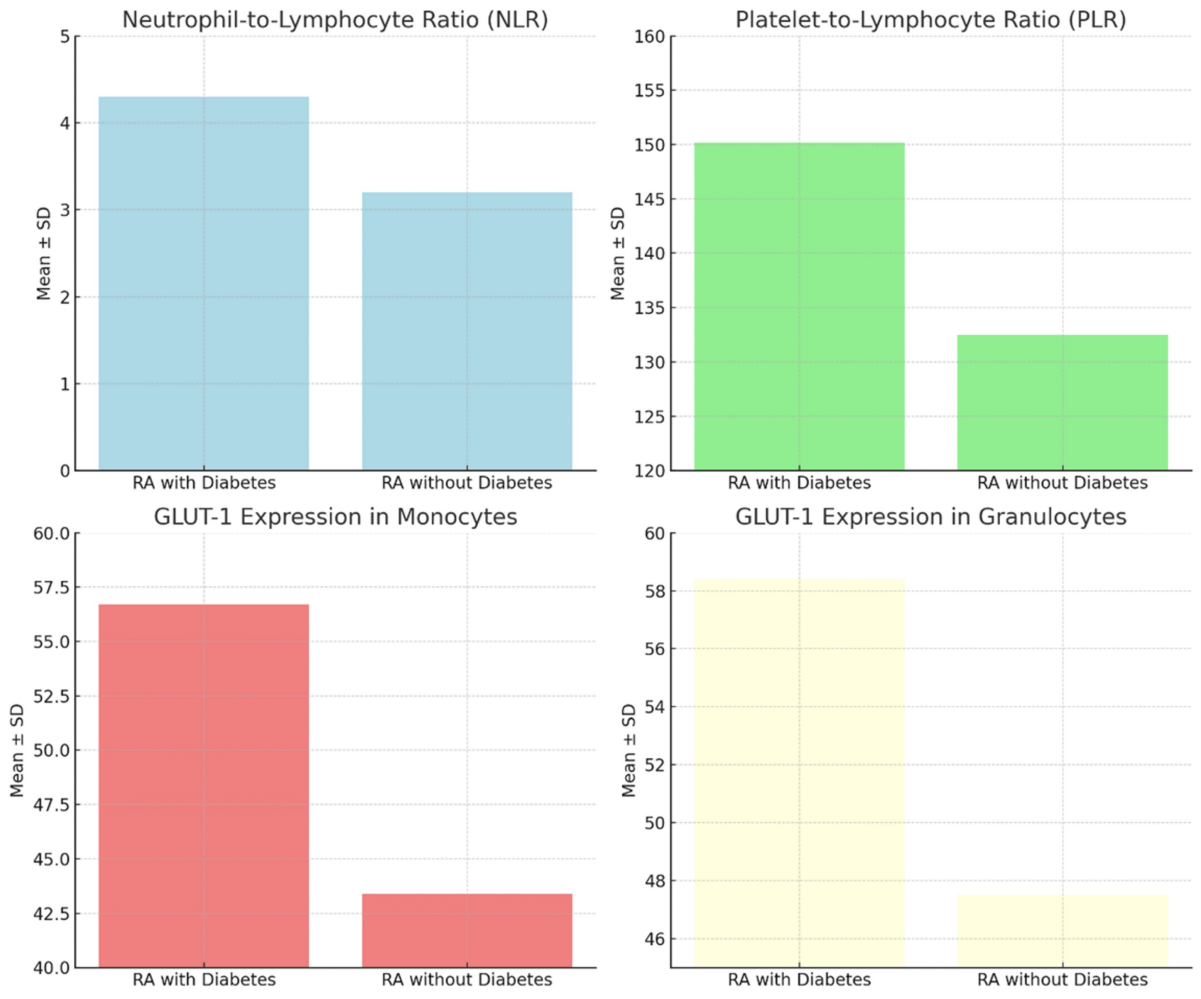
Neutrophil-to-lymphocyte ratio in RA patients. RA: rheumatoid arthritis

Compared with patients without metabolic involvement, individuals with rheumatoid arthritis and diabetes had a considerably higher neutrophil-to-lymphocyte ratio, according to an inflammatory index study. This observation is indicative of an exaggerated systemic inflammatory response among patients with insulin resistance. The identified difference demonstrates the utility of NLR as an easily available biomarker derived from standard hematology parameters. High NLR scores were consistently related to the group with metabolic alteration, which supports its possible use in identifying high-risk patients. Table 3 indicates that neutrophil-to-lymphocyte ratio values vary between the two groups of study participants, reflecting differences in systemic inflammation. Figure 1 shows a comparison of the NLR between rheumatoid arthritis patients with and without diabetes, indicating a higher inflammatory burden in the metabolically affected group.

**Table 3:** Comparison of neutrophil-to-lymphocyte ratio between study groups using the independent sample Student’s t-test. Data are presented as mean±SD. A p<0.05 was considered statistically significant. RA: rheumatoid arthritis

Groups	Mean±SD	t-value	p-Value
RA with diabetes	4.3±1.2	t=5.19	<0.001
RA without diabetes	3.2±0.9		

Platelet-to-lymphocyte ratio (PLR)

Patients with rheumatoid arthritis and insulin resistance had a significantly higher PLR than the non-diabetic patients. This finding also demonstrates that the metabolically compromised subgroup experiences increased systemic inflammation. The parameters of complete blood counts are simple and readily available, including inflammatory markers such as NLR, which are referred to as partial least square regression (PLSR). The high levels of PLR in the diabetic population can indicate that it could be used as an additional indicator of the inflammatory and metabolic overlap in RA. Table 4 presents a comparison of PLR in patients with rheumatoid arthritis, with or without diabetes, which suggests the presence of inflammatory variation.

**Table 4:** PLR comparison between RA groups using the independent Student’s t-test. Values are expressed as mean±SD. A p<0.05 was considered statistically significant. RA: rheumatoid arthritis; PLR: platelet-to-lymphocyte ratio

Groups	Mean±SD	t-value	p-Value
RA with diabetes	150.2±45.3	t=2.13	0.035
RA without diabetes	132.5±37.4		

GLUT-1 expression

Flow cytometric analysis revealed that there were significant elevations in the expression of GLUT-1 on circulating immune cells, and this was observed in patients with rheumatoid arthritis who have insulin resistance. Improved expression occurred, especially in monocytes and granulocytes, which pointed out the metabolic activity of inflammatory cells in the diabetic subgroup. GLUT-1 upregulation indicates a change in glucose metabolism, chronic inflammation, and insulin resistance. These data confirm the hypothesis that immune cell metabolic reprogramming can contribute to the inflammatory-metabolic interface in rheumatoid arthritis. Table 5 shows GLUT-1 expression levels in monocytes and granulocytes in both groups, illustrating the metabolic activation of immune cells.

**Table 5:** Comparison of leukocyte GLUT-1 expression between study groups was performed using the independent-samples Student’s t-test. Results are presented as mean±SD. A p<0.05 was considered statistically significant. RA: rheumatoid arthritis; GLUT-1: glucose transporter 1

Groups	Monocytes (mean±SD)	Granulocytes (mean±SD)	Test statistic	p-Value
RA with diabetes	56.7±13.2	58.4±14.1	t=5.54 (monocytes)	<0.001
RA without diabetes	43.4±10.7	47.5±11.2	t=4.27 (granulocytes)	<0.001

Insulin resistance and correlation with biomarkers

HOMA-IR was used to measure insulin resistance, which revealed a statistically significant difference between high levels in RA and diabetic patients. The correlational analysis showed that there is a significant positive association between inflammatory biomarkers and insulin resistance. NLR showed the best correlation compared to the GLUT-1 expression and PLR, which were the other examined parameters. These results reveal that there are strong interrelations between systemic inflammation and metabolic activation in patients with rheumatoid arthritis who are also insulin resistant. Insulin resistance and systemic inflammation are related, as evidenced by the association between HOMA-IR and inflammatory biomarkers (Table 6).

**Table 6:** Correlation between inflammatory biomarkers and insulin resistance was evaluated using Pearson’s correlation analysis (r). A p<0.05 was considered statistically significant. NLR: neutrophil-to-lymphocyte ratio; PLR: platelet-to-lymphocyte ratio

Biomarkers	Correlation coefficient (r)	p-Value
NLR	0.63	<0.001
PLR	0.48	0.01

Multivariate analysis

Multivariate linear regression analysis was performed to identify independent predictors of insulin resistance in patients with rheumatoid arthritis. After correcting for inflammatory factors, the independent predictors of HOMA-IR remained significant for NLR and monocyte GLUT-1 expression. The adjusted model did not retain the statistical significance of the expression of PLR and granulocyte GLUT-1. These data show that particular indicators of inflammatory and metabolic processes are independent predictors of insulin resistance in rheumatoid arthritis. Table 7 presents the multivariate regression model of independent predictors of insulin resistance among the assessed biomarkers.

**Table 7:** Multivariate linear regression analysis identifying predictors of insulin resistance (HOMA-IR). Results are expressed as standardized β coefficients with corresponding t-statistics and p-values. A p<0.05 was considered statistically significant. NLR: neutrophil-to-lymphocyte ratio; PLR: platelet-to-lymphocyte ratio

Predictors	β (standardized coefficient)	t-value	p-Value
NLR	0.42	t≈3.50	<0.001
PLR	0.13	t≈1.35	0.18

## Discussion

These current evidence-based results indicate that insulin resistance in rheumatoid arthritis (RA) is closely linked to increased systemic inflammation and immune cell metabolic activation. Nutritionally insulin-resistant RA patients displayed significantly higher NLR and platelet-to-lymphocyte ratio, and greater GLUT-1 expression, particularly in monocytic cells. Among these variables, NLR and monocyte GLUT-1 expression were independently predictive in multivariate analysis, highlighting their relative strength as indicators of metabolic disturbance in this group.

The rise in NLR reflects a shift toward innate immune dominance and relative lymphocyte suppression, both of which are characteristic of chronic inflammatory states. Neutrophils contribute to oxidative stress, cytokine amplification, and endothelial dysfunction, all of which interfere with insulin signaling pathways. The stronger predictive value of NLR compared to PLR supports a more direct mechanistic association between neutrophil-driven inflammation and insulin resistance in RA. Although elevated, PLR was not statistically significant, suggesting that platelet-associated inflammatory processes may be secondary or influenced by other hematologic or treatment-related factors.

The observed longer disease duration in patients with insulin resistance suggests a cumulative effect of chronic inflammation on metabolic pathways. Prolonged exposure to inflammatory cytokines may progressively impair insulin signaling, thereby increasing the risk of insulin resistance over time. This also introduces the possibility of duration-related bias, where patients with longer disease duration may inherently exhibit worse metabolic outcomes independent of other contributing factors.

Rheumatoid arthritis can be broadly classified into seropositive and seronegative subtypes based on the presence of rheumatoid factor (RF) and anti-cyclic citrullinated peptide (anti-CCP) antibodies. However, the absence of serological stratification represents a methodological limitation in the present study. Due to the unavailability of complete serological data for all participants, subgroup analysis based on RF and anti-CCP status could not be performed. This may have introduced heterogeneity, as seropositive patients typically exhibit a higher inflammatory burden, which could potentially overestimate the association between inflammatory markers and insulin resistance. Therefore, the findings should be interpreted with caution, and future studies incorporating serological stratification are necessary to validate these observations.

Seropositive patients are generally associated with higher disease activity, increased systemic inflammation, and poorer prognosis compared to seronegative individuals. This heightened inflammatory burden may contribute to metabolic disturbances, including insulin resistance. Although the present study did not stratify patients based on serological status, it is plausible that seropositive individuals may exhibit a greater degree of insulin resistance due to persistent inflammatory activation. Future studies incorporating serological classification are warranted to better elucidate this relationship.

The up-regulated expression of GLUT-1 on the monocytes or granulocytes provides the idea of the immunometabolic reprogramming in RA. Activated immune cells depend on increased glucose uptake to maintain glycolysis, cytokine secretions, and functions of the effector. GLUT-1 is up-regulated to enable this metabolic adaptation. Self-reported monocyte GLUT-1 and HOMA-IR are independent and thus indicate a biologically plausible mechanism of immunological activation leading to insulin resistance at the systemic level. Cytokine release and tissue infiltration of monocytes can have a direct role in adipose inflammation and the inhibition of peripheral insulin signaling. The latter example of the disease in the insulin-resistant subgroup also supports the fact that there is a cumulative inflammatory burden that contributes to metabolic dysfunction.

There are clinical and mechanistic implications of these findings. Both NLR and PLR are easily available as a result of standard blood counts, which makes them convenient in metabolic risk stratification of RA. The use of NLR as a routine measure can potentially uncover patients having a greater risk of insulin resistance and associated cardiometabolic complications, even though they would not be overtly obese or vastly dissimilar in disease activity indices. GLUT-1 expression is a more precise biomarker of stimulation of immune metabolism [16]. It needs a flow cytometric analysis, but its strong correlation with insulin resistance makes it potentially applicable to research and, perhaps, even to a specific clinical assessment [4]. Findings also justify the use of therapeutic interventions that focus on systemic inflammation, as well as immune metabolism, as a possible measure of alleviating insulin resistance in RA. Combinations of management models that deal with inflammatory control and metabolic monitoring can also decrease cardiovascular morbidity in the long term.

Past studies have always shown that high NLR is related to systemic inflammation, cardiovascular risk, and metabolic disorders, such as insulin resistance and type 2 diabetes [21]. In autoimmune diseases, an increase in NLR has been associated with increased disease activity and poor cardiometabolism [23]. These results are consistent with the existing outcomes, which positively determine NLR as an independent predictor of insulin resistance in RA. Data about PLR has been less homogeneous. Though there are studies indicating that elevated PLR is linked to inflammatory burdens or cardiovascular risks [13], there are also studies that depict weak or insignificant correlations with metabolic outcomes with multivariate adjustment [10]. The current results are not an exception to this trend, with PLR becoming non-significant in adjusted models.

Immunometabolism studies indicate that monocytes and other immune cells activated during chronic inflammation upregulate GLUT-1 expression to sustain glycolytic flux [19]. Such upregulation has been documented in autoimmune and metabolic diseases characterized by systemic inflammatory activation [24]. These observations support the biological plausibility of the association between monocyte GLUT-1 expression and insulin resistance observed in this study.

Inflammatory status in this study was assessed using the neutrophil-to-lymphocyte ratio (NLR), platelet-to-lymphocyte ratio (PLR), and glucose transporter 1 (GLUT-1). Conventional inflammatory markers such as erythrocyte sedimentation rate (ESR) and C-reactive protein (CRP) were not included. Although widely used in clinical practice, these markers may not fully capture the cellular and metabolic dimensions of inflammation. In contrast, NLR and PLR reflect dynamic immune cell balance, while GLUT-1 provides insight into immune-metabolic activity. However, the absence of ESR and CRP limits direct comparison with established inflammatory indices. Future studies should incorporate both traditional and novel biomarkers to enable a more comprehensive evaluation of inflammatory and metabolic interactions. Therefore, disease duration should be considered an important modifier when interpreting the relationship between inflammatory biomarkers and insulin resistance in rheumatoid arthritis.

NLR and PLR are simple, cost-effective hematological markers that reflect systemic inflammation and immune response. NLR primarily indicates the balance between innate (neutrophils) and adaptive (lymphocytes) immunity, while PLR reflects platelet activation in inflammatory states. GLUT-1, on the other hand, represents a molecular marker associated with increased glucose uptake in activated immune cells, linking inflammation with altered metabolic pathways. Compared to NLR and PLR, GLUT-1 may provide a more direct insight into the metabolic component of inflammation, particularly insulin resistance. The combined assessment of these markers offers a more comprehensive understanding of the interplay between inflammation and metabolic dysfunction in rheumatoid arthritis. This evidence is supported by the present findings, as they demonstrate an independent predictive correlation in RA, supporting the idea that immune metabolic reprogramming contributes to systemic metabolic dysfunction. The results are in agreement with the currently available literature, which reports the convergence of chronic inflammation, hematologic inflammatory indices, and immune cell metabolic activation in the pathogenesis of insulin resistance in inflammatory diseases.

Limitations and future recommendations

The primary limitation of this study is its cross-sectional design, which precludes establishing causal relationships among inflammatory biomarkers, glucose transporter 1 (GLUT-1) expression, and insulin resistance. Additionally, the moderate sample size and single-center setting may limit the generalizability of the findings. Insulin resistance was assessed using the homeostatic model assessment of insulin resistance (HOMA-IR), a practical and widely used method that incorporates fasting insulin and glucose levels, enabling detection of early or subclinical metabolic dysfunction; however, it remains an indirect measure and is less precise than gold-standard clamp-based techniques. The absence of adjustment for potential confounding factors, including diet, physical activity, and medication use, may have introduced residual bias, as these variables can influence both inflammatory status and metabolic outcomes. Furthermore, conventional inflammatory markers, such as the erythrocyte sedimentation rate and C-reactive protein, were not included, limiting comparison with established clinical indices. The lack of stratification based on serological status (seropositive vs. seronegative) may also have introduced heterogeneity, as differing inflammatory profiles could affect metabolic outcomes. Collectively, these limitations should be considered when interpreting the findings.

## Conclusions

The study shows that rheumatoid arthritis patients with insulin resistance exhibit a close correlation with systemic inflammation and immune cell metabolic activation. NLR and PLR were significantly higher in patients with rheumatoid arthritis and insulin resistance, and the GLUT-1 expression on circulating immune cells, especially monocytes and granulocytes, was significantly increased. Among the considered markers, NLR and monocyte GLUT-1 expression have emerged as the most predictive independent variables for insulin resistance, making them potentially useful in a clinical setting for the early prediction of patients with rheumatoid arthritis who may be at risk of metabolic conditions. The fact that HOMA-IR was positively correlated with these biomarkers supports the idea that chronic inflammatory responses and changes in immune glucose utilization of biomarkers play a role in the metabolic dysregulation in RA. NLR and PLR are straightforward and readily available measures that can be used as convenient screening tools in daily practice, and GLUT-1 expression can be used to understand the mechanistic aspects of immunometabolic pathways in which inflammation relates to insulin resistance. The results contribute to the significance of combined inflammatory and metabolic evaluation in rheumatoid arthritis and contribute to the further study of specific measures aimed at minimizing cardiometabolic risk in this group.
